# Larvicidal potential, antimicrobial properties and molecular docking analysis of Egyptian Mint (*Mentha rotundifolia*) against *Culex pipiens* L. (Diptera: Culicidae) and Midgut-borne *Staphylococcus aureus*

**DOI:** 10.1038/s41598-024-51634-2

**Published:** 2024-01-19

**Authors:** Samah H. Abu-Hussien, Bahaa Hemdan, Basma T. Abd-Elhalim, Mohamed M. Aboul Fotouh, Ahmed G. Soliman, Youssef K. Ghallab, Eslam Adly, Salwa M. El-Sayed

**Affiliations:** 1 Department of Agricultural Microbiology, Faculty of Agriculture, Ain Shams University, Cairo, 12411 Egypt; 2 Water Pollution Research Department, Environmental Research and Climate Change Institute, National Research Centre, 33 El-Bohouth St., Dokki, Giza, 12622 Egypt; 3 Department of Agriculture Biochemistry, Faculty of Agriculture, Ain Shams University, Cairo, 12411 Egypt; 4 Biotechnology program, New Programs, Faculty of Agriculture, Ain Shams University, Cairo, 12411 Egypt; 5 Department of Entomology, Faculty of Science, Ain Shams University, Cairo, 11566 Egypt

**Keywords:** Biochemistry, Microbiology, Entomology

## Abstract

Mosquitoes prefer stagnant areas near hospitals to live and easily spread pathogenic bacteria. Our current study aims to isolate multidrug-resistant (MDR) *Staphylococcus aureus* isolates from midguts of Mosquito *Culex pipiens* and study the potential of mint as a biocontrol strategy against *C. pipiens* larvae and their midgut-borne *S. aureus*. Samples of the third and fourth larval instars of *C. pipiens* were collected from water ponds around three Cairo hospitals. Ciprofloxacin, gentamycin and tetracycline, as well as various concentrations of mint leaf extract (MLE) were tested for antibiotic susceptibility. Sixty-five isolates were obtained and showed antibiotic resistance to tetracycline, gentamycin, ciprofloxacin, and undiluted MLE with resistant percentages (%) of 27.69, 30.76, 17.46, and 23.08%, respectively. Undiluted MLE inhibited 61.53% of the multidrug *S. aureus* isolates, whereas it couldn't inhibit any of these isolates at dilutions less than 50 μg/mL. The MIC of MLE was ≤ 700 µg/mL, while the MIC of the antibiotics ranged from 0.25 to 5.0 µg/mL for the three antibiotics. The most inhibited *S. aureus* isolate was identified by 16SrRNA sequencing approach and registered in GenBank as *S. aureus* MICBURN with gene accession number OQ766965. MLE killed all larval stages after 72 h of exposure, with mortality (%) reaching 93.33 and 100% causing external hair loss, breakage of the outer cuticle epithelial layer of the abdomen, and larvae shrinkage. Histopathology of treated larvae showed destruction of all midgut cells and organelles. Gas chromatography (GC) of MLE revealed that menthol extract (35.92%) was the largest active ingredient, followed by menthone (19.85%), D-Carvone (15.46%), Pulegone (5.0579%). Docking analysis confirmed that alpha guanine and cadinol had the highest binding affinity to both predicted active sites of *Culex pipiens* acetylcholinesterase. As a result, alpha-guanine and cadinol might have a role as acetylcholinesterase inhibitors.

## Introduction

Insects are chronically colonized with a wide variety of gut microflora that is well known to cause many human diseases. Both the insects' midguts and their whole bodies carry different microflora that helps their host's development. On the other side, this microflora could be transmitted to humans through different transmission means and cause several illnesses and diseases^1^. The common house mosquito, *C. pipiens* L. (Culicidae), is considered the superlative historical lymphatic filariasis vector in Egypt and the world, as reported by Ding et al. ^2^. *C. pipiens* is well known as an insect vector that indeed threatens human public health due to their ability to transmit many causative agents such as arboviruses: Flaviviruses (Flaviviridae), pleboviruses (Phenuiviridae)*, worms (Wuchereria bancrofti*, Onchocercidae), and *Plasmodium relictum* (Plasmodiidae) that cause many diseases like West Nile fever, Japanese encephalitis, Dengue fever, Rift Valley fever, Bancroftian filariasis, and Avian malaria.

Many researchers have studied the vectorial capacity of *Culex* sp. toward virus particles, but there is a gap in knowledge about their gut microbiota, especially their midgut-borne bacteria and fungi^3^. The transmission of bacterial foodborne pathogens like *Bacillus cereus*, *Bacillus anthrax*, and *Staphylococcus warneri* has recently been reported by *C. pipiens*, despite the lack of knowledge regarding the gut bacterial symbionts of this organism and its widespread existence^4^.

The term "ESKAPE" includes six highly virulent and multidrug-resistant (MDR) pathogens. They include the non-hemolytic G^+ve^ bacteria (*Enterococcus faecium* and *Staphylococcus aureus*) and the G^−ve^ bacteria (*Klebsiella pneumoniae*, *Acinetobacter baumannii*, *Pseudomonas aeruginosa*, and *Enterobacter cloacae*)^5^. *S. aureus* is one of the main causative agents for nosocomial infections, especially wound infections in hospitalized patients, and it is responsible for the morbidity and mortality of many infected individuals^6^. The illnesses caused by *S. aureus* infection include urinary, wound, and nosocomial infections, as well as bacteremia and endocarditis. These infections could accelerate death, especially for immune-compromised patients with cystic fibrosis, debilitated patients, and worn-wound patients^7^.

Rates of antibiotic resistance are increasing worldwide due to continuous exposure and the misuse of antibiotics^8^. *S. aureus* becomes difficult to eradicate using several antibiotics. This resistance is due to its high potential for resistance against many different commercial antibiotics. *S. aureus* uses many resistance mechanisms, such as inherent, acquired, and genetic resistance. Additionally, it generates a significant number of enzymes that function as antibiotic inhibitors, resulting in antibiotic resistance^9^. Inducible lactamase, one of the enzymes they produce to inhibit antibiotics, is essential for the development of unstable resistance^10^. It is so important to face the rapid increase of MDR *S. aureus* in hospitalized patients^11^.

Generally, herbal plants are widely considered lately in therapeutic strategies to control *S. aureus* because of their high composition of biologically active compounds^1^. The Lamiaceae family, which has more than 350 known plant species, includes Egyptian mint (*Mentha rotundifolia*), one of the most popular medicinal plants^12^. Mint can be used as an alternative antimicrobial and anti-inflammatory agent to harmful synthetic medicines; moreover, it is used in the food processing and cosmetics fields. Mint has a long history of use in the agricultural, cosmetics, public health, and traditional medicine industries^13^. Therefore, the purpose of this study is to investigate MLE's larvicidal and antimicrobial effects on *C. pipiens* larvae and the *S. aureus* that is carried in their midgut.

## Materials and methods

### Chemicals and reagents

Phosphate buffer, Mueller–Hinton, and Baird Parker agar media were purchased from Sigma, Aldrich Germany. Tryptic soy broth were obtained from Oxoid, England. Isopropanol was purchased from analar, BDH, England. Ciprofloxacin, gentamicin, and tetracycline antibiotic discs were commercially obtained from the Novartis, Pharco, and Amoun companies in Cairo, Egypt. All chemicals are analytical grades.

### *C. pipiens* larvae collection

Third and fourth larval instar *Culex pipiens* L. larvae samples were collected randomly between November 2022 and February 2023 from three water ponds near three general hospitals in the Cairo governorate of Egypt: Dar El-Salam General Hospital, Road El Farag General Hospital, and ElZawya ElHamra General Hospital. All the collected larvae were put in sterilized glass jars and brought to the preparations lab. located in Microbial Inoculant Center, Faculty of Agriculture, Ain Shams University, Cairo, Egypt.

### Isolation of mid-gut-borne *S. aureus* in *C. pipiens* larvae

To isolate *S. aureus*, 3rd and 4th instar mosquito larvae were surface sterilized by soaking in 70% ethyl alcohol for 30 s, followed by 5 washes in sterile Milli-Q water to remove excess ethyl alcohol. Under aseptic conditions, the heads and abdomens of the larvae were removed using a sterile scalpel and forceps. The extracted midguts were placed in 100 mL phosphate-buffered saline (PBS). The midguts were homogenized in 100 mL PBS using a sterile plastic pestle. The homogenates were inoculated onto Parker agar plates supplemented with 0.01% (w/v) potassium tellurite to select for *S. aureus*. Plates were incubated at 37 °C for 24–48 h. Rough, black colonies displaying yellow zones of precipitation, showing mannitol fermentation, were presumptively identified as *S. aureus*. Isolates were subcultured on Tryptic soy agar to get pure cultures for downstream testing. Antibiotic susceptibility testing was performed on the isolates using commercially available ciprofloxacin, gentamicin, and tetracycline antibiotic discs and undiluted MLE extract^15^.

### Mint leaf extraction

The leaves of mint (*Mentha rotundifolia*) (Family: Lamiaceae) were purchased from a local market in Cairo, Egypt. The leaves were washed twice with tap water, followed by a rinse with distilled water to remove minerals and chlorine. The leaves were then dried and ground in an electrical grinding machine (Molineux, AR6801EG). For storage, the harvested mint powder was placed in airtight jars. For extraction, mint powder (10 g/100 mL) was added to isopropanol, followed by a filtration process after 6 h. To avoid contamination, the collected filtrate was evaporated in a water bath at 60 °C. The extract was filter-sterilized after evaporation of the isopropanol. The sterilized extract was then added to sterilized bottles sealed with parafilm^16^. MLE extract was dissolved in DMSO and then further diluted in broth.

### Antibiotic susceptibility test for MLE and the three commercial antibiotics against *S. aureus* isolates

Antibiotic susceptibility testing was performed using the disc diffusion method following CLSI guidelines. Commercially antibiotic discs containing ciprofloxacin (5 μg/disc, gentamicin (10 μg/disc), and tetracycline (25 μg/disc) were utilized at the standard dose concentrations for antibiotic susceptibility testing, as they are commonly used to treat *S. aureus* infections. A control *S. aureus* ATCC 29737 strain was used as a positive control. Isolates and controls were inoculated onto Mueller–Hinton agar plates and incubated at 37 °C for 24 h. Inhibition zone diameters (IZD) in cm were measured and interpreted using CLSI breakpoints to determine susceptibility. Experiments were done in triplicate. The most resistant isolates were selected for further characterization. Multidrug resistance was defined as resistance to at least two antibiotics. The isolate with the highest resistance was chosen for additional studies. This approach identified antibiotic-resistant *S. aureus* isolates from the mosquito larvae samples^15^. The identity of the selected *S. aureus* isolate was definitively confirmed through molecular identification, as described in the subsequent section.

### Minimum Inhibitory Concentration (MIC) of the commercial antibiotics

All *S. aureus* isolates were subjected to an antimicrobial susceptibility test using ciprofloxacin (5µg/mL), gentamicin (10µg/mL), and tetracycline (25 µg/mL) by the micro-dilution method, following the recommendations of the Clinical Laboratory and Standards Institute (CLSI)^15^. The MIC was calculated and defined as the antibiotic's lowest concentration that inhibited all the visible growth of each tested isolate. After calculating the MIC, all dilutions were added to plates to count the total number of bacteria and determine the minimal bacterial concentration (MBC). MBC was defined as the lowest antibiotic concentration at which no colonies were recorded. The most resistant isolate*, S. aureus* S35, was selected for further studies.

### Minimum Inhibitory Concentration (MIC) of MLE against *S. aureus* isolates

The mint stock solution was prepared in tryptic soy broth. pH was adjusted to 7.2 at 25 °C. Different concentrations of MLE extract were prepared at 800, 700, 600, 500, 400, 300, 200, 100, 50, and 25 mg/mL by diluting the stock extract in broth to achieve the desired concentrations for susceptibility testing. 200 μg/mL of each dilution was added to 96-well cell culture plates. A 100 μL of the most resistant *S. aureus* isolate suspension was added to each well and incubated at 37 °C for 24 h. For negative controls, wells were filled with broth only with no MLE extract or antibiotic exposure. Absorbance was measured at 595 nm. As stated earlier, estimates for MIC and minimal bacterial concentration (MBC) were carried out.

### Molecular identification for the most susceptible *S. aureus* isolates

A QIA amp DNA mini kit from QIAGEN GmbH, Hilden, Germany, was used to extract the DNA from the pure culture of the incredibly sensitive *S. aureus* isolate following the manufacturer's instructions. The 16S rRNA gene sequences were targeted by the universal primers 27F (5′ AGAGTTTGATCCTGGCTCAG 3′) and 1492R (5′ TACG GCTACCTTGTTACGACTT 3′). The nucleotide FASTA sequence was submitted to the NCBI GenBank under accession number (OQ766965) and NCBI database BLAST (http://www.ncbi.nlm.nih.gov/BLAST). Mega 11 software was used to view the distance tree for the sequences, and it was then used to build a neighbor-joining phylogenetic cladogram tree with identification based on sequence similarity^17^.

### MLE's impact on the mortality of *C. pipiens* larvae

For the current study, 25 larvae of each size (3rd to 4th instar stage) were placed in 100 mL beakers containing various concentrations of previously prepared MLE. The MLE was initially dissolved in 10% DMSO to prepare MLE stock solution (1 mg/mL). Different testing concentrations of MLE (100%, 75%, 50%, 25%, 10%, and 0%) were then prepared by diluting the MLE stock solution in water. The effect of MLE on *C. pipiens* larvae's viability was determined for all concentrations using Breadcrumbs as nutrients. All larvae were incubated at 25–30 °C under a 14:10 light and dark photoperiod cycle for 72h. The total larval time (days) was calculated from the first day of treatment until the death day of the larvae. The number of dead larvae in each batch was counted every day in the morning. The negative control of each experiment (treated with DMSO-distilled water) was tested three times. The mortality (using Abbott's formula) and survival rate were determined after 24, 48, and 72 h of exposure. All the test containers were kept at room temperature with no disturbance. Correct for mortality in the control treatment using Abbott's formula (% test mortality -% control mortality/100—control mortality × 100)^18^.

### Morphological and histopathological studies of *C. pipiens*

Based on the MLE’s impact on *C. pipiens* mortality result, the morphological changes of the treated larvae were determined using a Labomed microscope (Labomed® Microscopes Manufacturers & Suppliers, Labo America Inc.) (at 40 and 100 ×). The dead larvae were counted on a microscopic slide and observed under the microscope^19^. For microscopic examination, all control and treated larvae were fixed with 3–5% formalin, then dried out using ethyl alcohol and cleaned with xylene. All prepared slide samples were fixed with a paraplast to take sections (7 µm). Eosin and hematoxylin stains were used for staining the sectioned larvae and control treatment^20^. The mid-guts of control and treated larvae were examined and photographed using a labomed microscope.

### Chemical determinations

All chemical analyses were performed in Nawah Scientific Labs (www.nawah-scientific.com), Mokattam branch, Cairo, Egypt.

### Total protein content

Total protein content was determined using a BCA assay kit (ThermoScientific). 50 μL of protein standards and larval samples were added to tubes along with 450 μL water, 100 μL of 0.15% sodium deoxycholate, and 100 μL of 72% trichloroacetic acid to precipitate proteins. After centrifugation at 10,000 rpm for 15 min and removal of the supernatant, 50 μL of 5% SDS reagent was added to dissociate the precipitated proteins. 1 mL of BCA reagent was then added, which reacts with protein peptides to form a purple-colored complex measured at 562 nm. Absorbance was compared to a standard curve to calculate total protein levels per 25 larval samples. The assay involves precipitating proteins out of solution, and then resolubilizing them to allow colorimetric detection as a measure of total protein content^21^.

### Total carbohydrates content

For quantification of total carbohydrates, the glucose standard for total soluble carbohydrates assay was carried out^21^. Briefly, a glucose stock solution of 1 mg was dissolved in 1 mL of distilled water, from which the following concentrations were prepared: 400, 200, 100, 50, 25, and 12.5 µg/mL. Larvae samples were homogenized using a sterilized mortar and then centrifuged at 10,000 rpm. Pellets were discarded, and the supernatant was collected and diluted by a ratio of 1:1 in distilled water. A 100 µL of concentrated sulfuric acid solution (75% v/v) was added to 50 µL of larvae sample in a glass vial. The vial was then filled with 200 µL of the anthrone reagent (5 mg in 100 µL of ethanol and 2.4 mL of 75% v/v sulfuric acid), and the temperature of the oven was set at 100 °C for 5 min. The mixture was heated, and allowed to cool for five minutes at room temperature, and then the analysis was performed by transferring 100 L of the sample mixture to a 96-well plate (n = 6, three independent experiments), where the resulting green color was measured at 578 nm. Data are represented as means ± SD. The results were recorded using a FluoStar Omega microplate reader^22^.

### Acetylcholine esterase activity

Donepezil standard was prepared at the concentration of 5 mM to serve as a positive control. Larvae samples were prepared as mentioned before. Acetylcholinesterase enzyme was purchased from Sigma-Aldrich from the Electrophorus electrics. Cat number: 3389. Aceylthiocholine iodide substrate and the indicator 3,3′-Dithiodipropionic acid di (N-hydroxysuccinimide ester) (DTNB) were purchased from Sigma-Aldrich. Briefly, 10µL of the indicator solution (0.4 mM in buffer (1): 100 mM tris buffer pH-7.5) was transferred to a 96-well plate followed by 20µL of enzyme solution (acetylcholine esterase 0.02U/mL final concentration in buffer (2): 50 mM tris buffer PH = 7.5 containing 0.1% bovine serum albumin). Following that, 140 mL of buffer was added, then 20 mL of the sample/standard solution (1). The mixture could stand for 15 min at room temperature. The substrate (0.4 mM acetylcholine iodide buffer (1)) was then added to each well in an instant, totaling 10 L. The plate was incubated in a dark chamber for 20 min at room temperature. At the end of the incubation period, the color was measured at 412 nm. Data are represented as means ± SD. The results were recorded using a microplate reader, FluoStar Omega^21,22^.

### GC-Mass chromatography

MLE was dissolved using 3 mL ethyl acetate and 1 mL was transferred to GC vial for GC/MS analysis after evaporation. The investigation of various substances of MLE that were present in modest quantities was performed using gas chromatography-mass spectrometry. The components were identified by comparing their mass spectra and retention times to those of the authentic compounds, as well as by computer matching with the NIST and WILEY libraries and by comparing the fragmentation pattern of the mass spectral data to those reported in the literature. The analysis was performed with a GC (Agilent Technologies 7890A, Poway, CA) interfaced with a mass-selective detector (MSD, Agilent 7000, Poway, CA) equipped with a polar Agilent HP-5 ms (5%-phenyl methyl poly siloxane) capillary column (30 m 0.25 mm in diameter and 0.25 m film thickness). Helium was used as the carrier gas, with a linear velocity of 1 mL/min. The injector and detector temperatures were 200 °C and 250 °C, respectively. Volume injected was 1 μL of the sample. The MS operating parameters were as follows: ionization potential 70 eV, interface temperature 250 °C, and acquisition mass range 50–800^23^.

### Acetylcholine esterase and Beta-lactamase binding interactions by molecular docking

Acetylcholinesterase from *C. pipiens* and Beta-lactamase from *S. aureus* were the two proteins examined in the study, along with their associated ligands. The X-ray crystal structure of acetylcholinesterase from *Drosophila melanogaster* (PDB ID 1QON) and a homology model of acetylcholinesterase from *C. pipiens* were obtained from the RCSB Protein Data Bank and SwissModel repository respectively. These existing insect AchE structures were used as targets for docking. The proteins' 3D model structures were obtained from the UniProt KB database, improved using ModRefiner to increase the protein quality, and their active sites were predicted using Deepsite^24^. The ligands' structures were retrieved from the PubChem database, and their 3D structures were energy-minimized using Avogadro 1.2.0 software^25^. Docking simulations were performed using AutoDock Vina software with a grid box size of 20 × 20 × 20, and molecular dynamics simulations were generated using the MOE 2015 software and the SIBioLead web server. ADMET analysis was performed using the ADMETlab 2.0 web server^26^, and pathway analysis was conducted using the STITCH database. Finally, a 3D-QSAR analysis was performed using the Cloud 3D-QSAR web tool by integrating the SMILES codes for each compound with a pIC_50_ value.

### Statistical analysis

All samples and collected data were statistically analyzed using IBM® SPSS® Statistics software (2017). A Tukey test was conducted with a P-value of 0.05^27^.

### Ethical statement

This article does not contain any studies with human participants or animals performed by any of the authors. It was approved by the ethical committee of Faculty of Agriculture, Ain Shams University, Cairo, Egypt.

## Results

### Antimicrobial potential of commercial antibiotics against clinical *S. aureus* isolates

**Figure 1:**
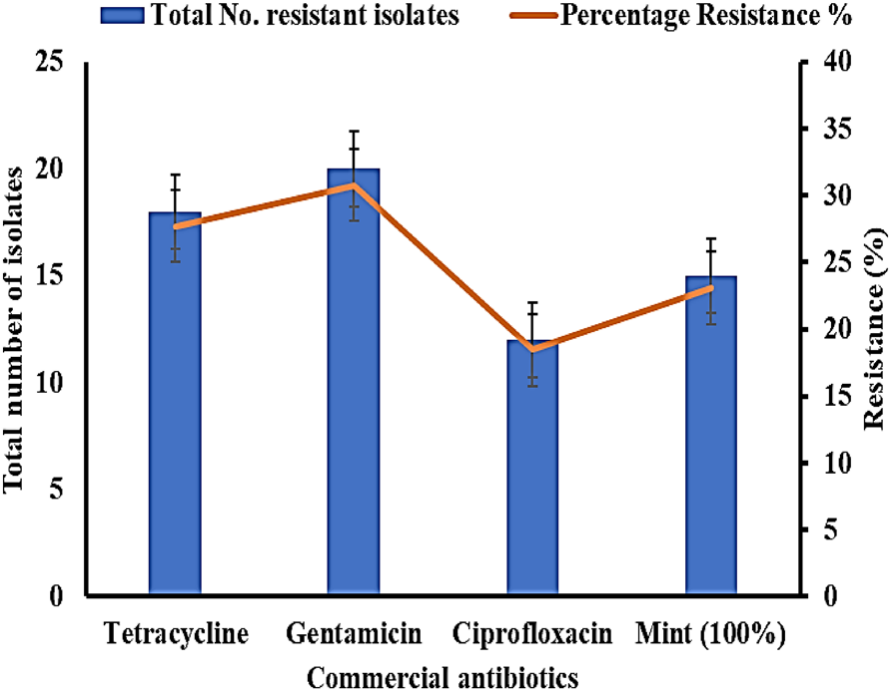
Multidrug resistance (MDR) of *S. aureus* isolates collected from *C. pipiens* midguts.

In the current study, 65 *S. aureus* isolates were gathered from field larvae midgut samples that were collected near three hospitals in Cairo, Egypt. A susceptibility test was performed using the disc diffusion method. Of the 65 collected isolates, 18 (27.69%) were resistant to tetracycline, 20 (30.76%) were resistant to gentamycin, 12 (17.46%) were resistant to ciprofloxacin, and fifteen (23.08%) were resistant to MLE, as illustrated in Fig. 1. As shown in Table 1, MLE's inhibitory activity was recorded as inhibition zone diameter (IZD) expressed in milliliters. Antibiotic susceptibility testing was performed against a control *S. aureus* strain and the selected *S. aureus S35* isolate. With recorded inhibition zone diameters ranging from 2.4 to 5.2 cm, *S. aureus* isolate S35, one of the clinical isolates obtained from the mosquito larvae, was identified as the most resistant to the undiluted MLE out of all the isolates tested. Inhibition zone diameters (IZD) in cm were measured to assess the degree of growth inhibition. The control *S. aureus* strain displayed susceptibility to the antibiotics and mint extract with larger IZDs. The isolate S35 showed reduced IZD sizes, indicating decreased susceptibility and antimicrobial resistance. The results demonstrate the potential antibacterial activity of mint extract against *S. aureus* but also reveal challenges with antibiotic resistance strains.

**Table 1:** Antibiotic susceptibility test for MLE and the three commercial antibiotics against *the S. aureus* S35 isolate and the positive control strain *S. aureus* ATCC 29737.

Antibiotic	Conc. (µg/disc)	Inhibition zone (cm)			
		*S. aureus* ATCC 29737	IZD	*S. aureus* S35	IZD
Ciprofloxacin(CIP)	5.00	4.23 ± 1.27^b^	S	2.34 ± 0.06^e^	S
Gentamicin (GEN)	10.0	3.19 ± 0.10^e^	I	1.40 ± 0.10f	I
Tetracycline (TE)	10.0	5.40 ± 0.15^a^	S	2.23 ± 0.12^e^	I
Mint (100%)		5.20 ± 0.15^a^	S	2.46 ± 0.21^d^	I

IZD = Inhibition zone diameter, Conc. = concentration, R = Resistant, I = intermediate, S = sensitive. All values are the mean of three replicates ± standard deviation (SD) according to Tukey’s test at confidence 5^27^.

### Inhibitory activity of MLE against multidrug-resistant *S. aureus* isolates

**Figure 2:**
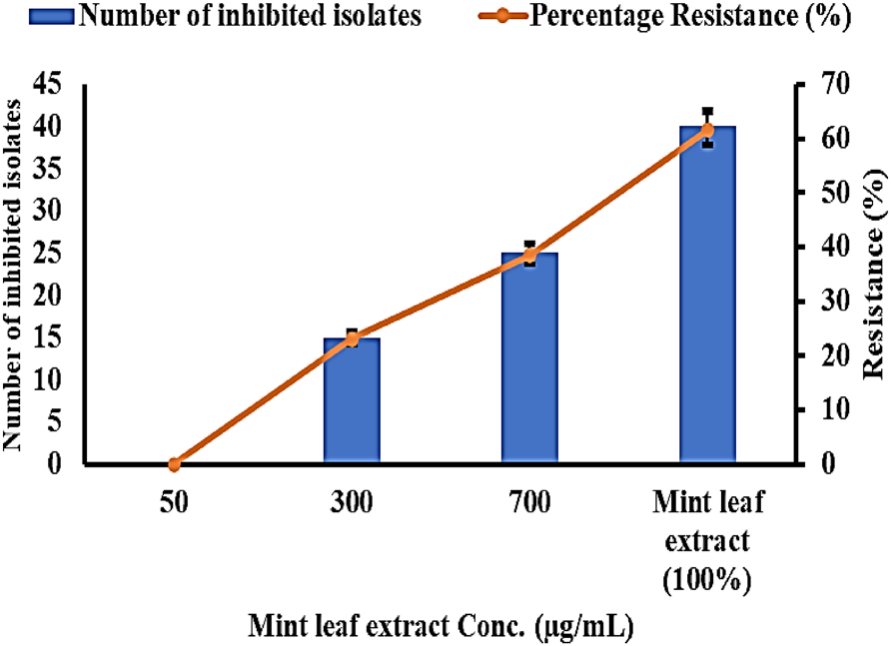
Inhibitory activity of mint concentrations against all *S. aureus* isolates collected from *C. pipiens* midguts.

The results showed that undiluted MLE could inhibit a subset of the multidrug-resistant *S. aureus* isolates. Specifically, MLE at concentrations of 300 and 700 μg/mL inhibited 35 out of the 45 multidrug-resistant isolates tested, representing 77.77% resistance**.** According to data in Fig. 2, undiluted mint inhibits 61.53% of the multidrug-resistant *S. aureus* isolates. However, Mint could inhibit none of these isolates at dilutions ≤ 50 μg/mL.

### Minimal inhibitory concentrations (MIC) for antibiotics and MLE

**Figure 3:**
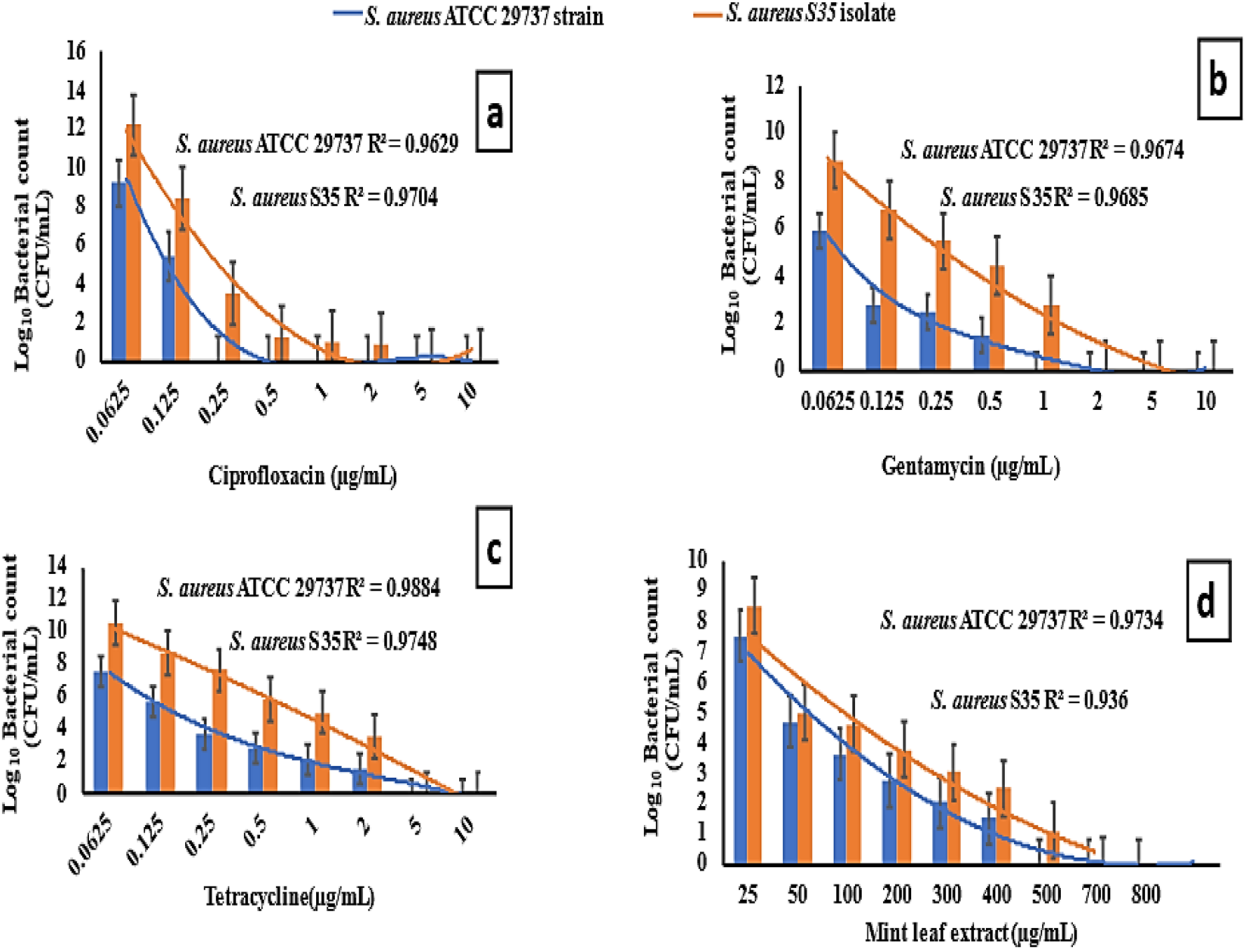
MIC of the tested three antibiotics (ciprofloxacin, gentamycin, and tetracycline) and MLE against *S. aureus S35* isolate and *S. aureus* ATCC 29737 as positive control.

MIC value of the antibiotics and mint leaf extract (MLE) was determined against the *S. aureus* isolates using a microdilution assay, Fig. 3. *S. aureus* ATCC 29737 served as a positive control. The results showed isolate S35 was susciptable to the antibiotics at concentrations of 0.25–5 μg/mL. In contrast to ciprofloxacin, which had a MIC of 0.25 μg/mL for isolate S35, MLE had a higher MIC of 700 μg/mL. This demonstrated the relative efficacy of the antibiotics compared to MLE, with the antibiotics being effective at much lower concentrations than the mint extract.

### Molecular identification of the selected *S. aureus* S35 isolate

**Figure 4:**
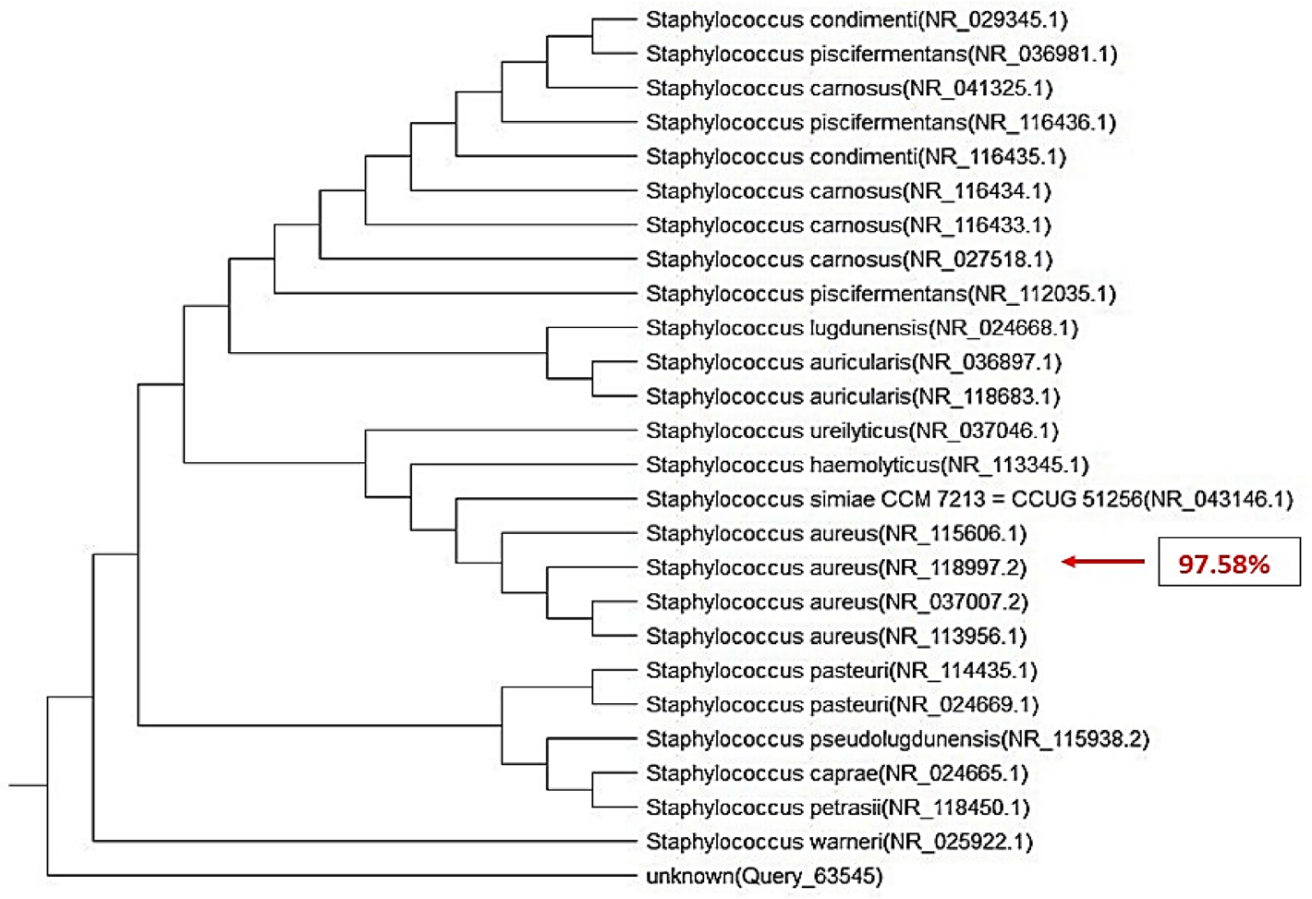
The phylogenetic tree of 16S rRNA gene sequences of *S. aureus* MICBURN as compared to 25 strains recorded in GenBank with gene accession number OQ766965.

The 16S rRNA sequence was amplified using a universal primer, yielding an amplified product of 1500 bp. The NCBI obtained this sequence and used the BLASTN program to compare it to the Gen Bank databases. (https://www.ncbi.nlm.nih.gov/), Fig. 4. A similarity percentage revealed a close relatedness to the 97.58% similarity of *S. aureus* NR_118997.2. Hence, the strain was confirmed as *S. aureus* MICBURN with gene accession number OQ766965.

### Effect of MLE on the Mortality of *C. pipiens* larvae

**Figure 5:**
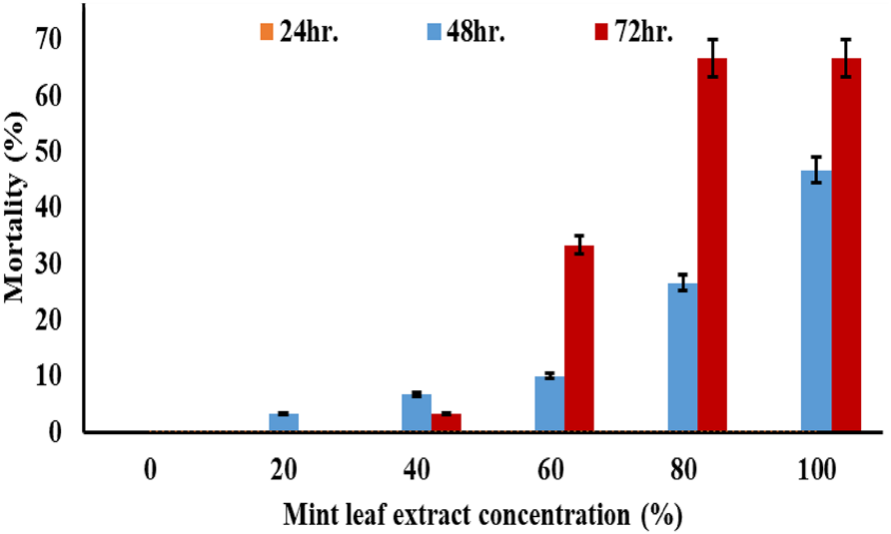
The mortality percentage (%) of *C. pipiens* larvae in their third and fourth instars after a 72-h incubation period at 25 °C is affected by MLE concentrations (0, 20, 40, 60, 80, 100%).

The larvicidal effect of MLE on *C. pipiens* larvae was demonstrated in Fig. 5. Gradually raising the concentrations of the mint extract had a lethal (larvicidal) effect that, after 72 h, was rated from low to moderate (or slightly high), reaching 68%. After exposure for 72 h, 100% of MLE experienced 100% mortality, indicating a particularly strong effect at that point. After exposure for 72 h, 100% of MLE experienced 100% mortality, indicating a particularly strong effect at that point.

### Morphological changes of *C. pipiens* larvae treated with MLE

**Figure 6:**
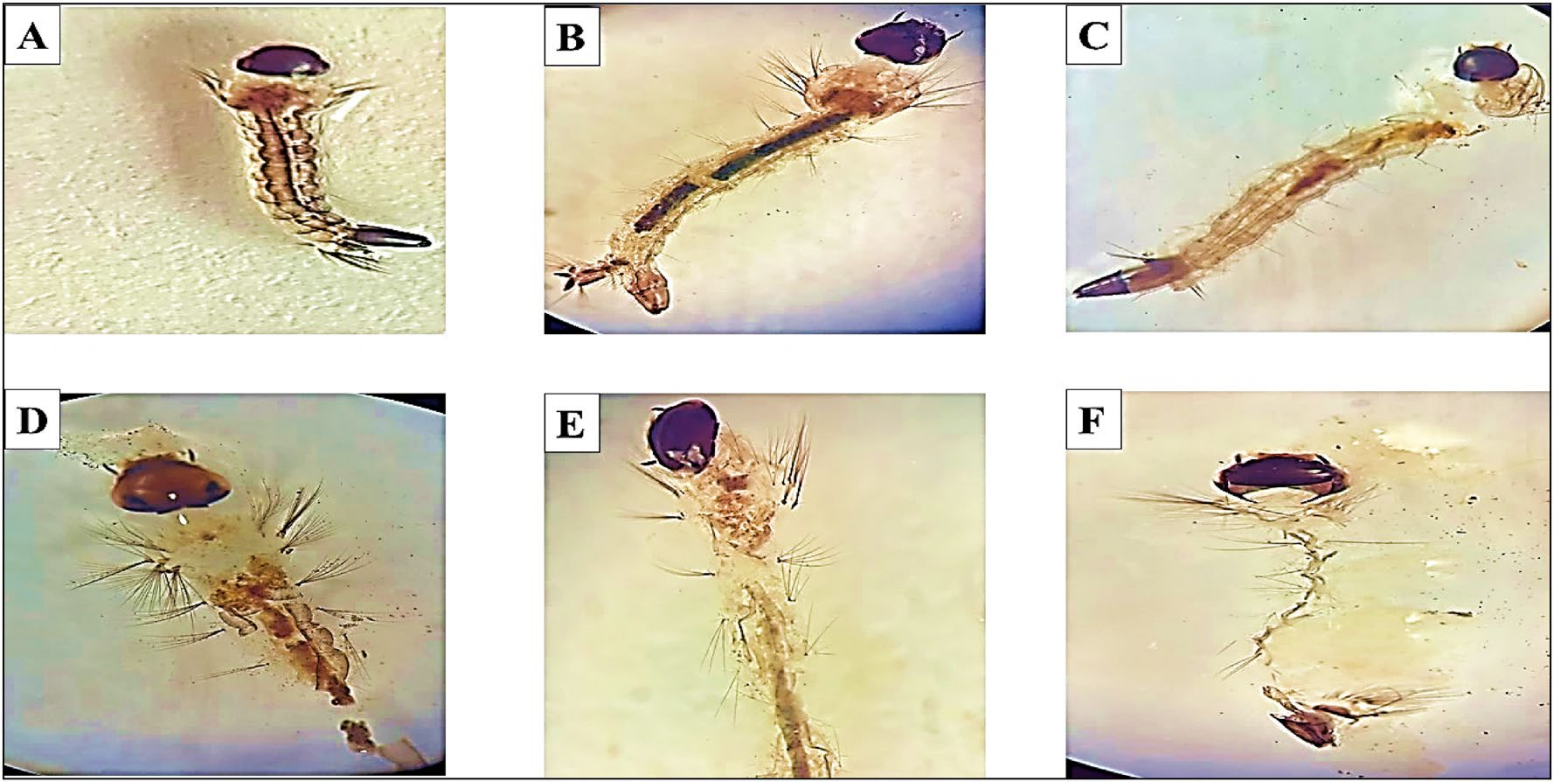
The effects of MLE concentrations (0, 20, 40, 60, 80, 100%) on the morphological traits of third and fourth instar *C. pipiens* larvae incubated for 72h at 25 °C. (A: control, B: 20%, C: 40%, D: 60%, E: 80%, F: 100% of MLE concentrations).

The third and fourth instars of control larvae of *C. pipiens* exposed to MLE for 72 h (A) show microscopic changes in the head, thorax, midgut, and anal gill parts (B-F). The toxic effects of 20–100% MLE on *C. pipiens* include loss of external hairs, the epithelial layer's outer cuticle crumbling, abdominal breakage, and larval shrinkage in addition to toxic effects on various body parts (thorax, midguts, and anal gills) (Fig. 6).

### Histological modifications for MLE-treated

**Figure 7:**
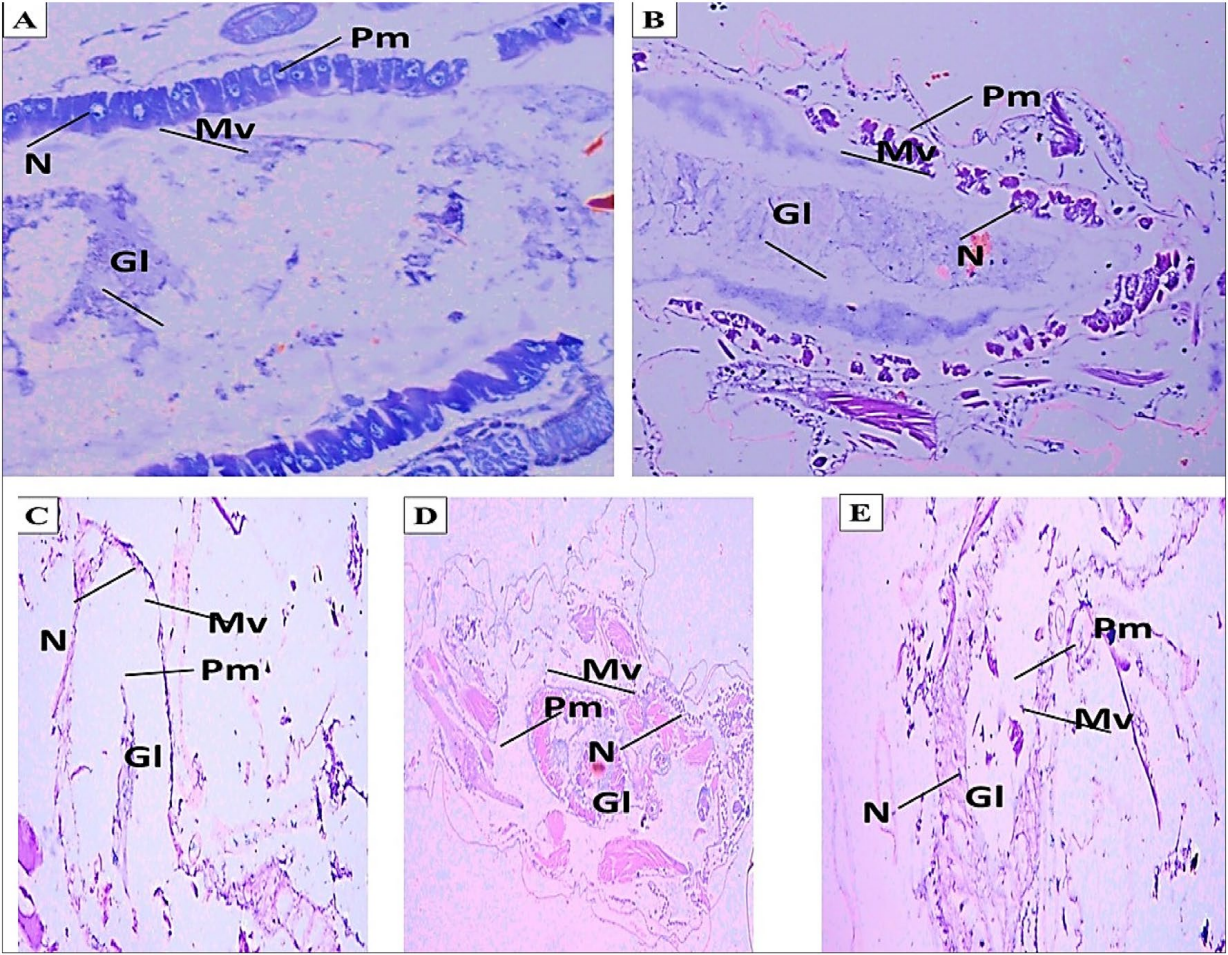
Histopathology malformations of larvae treated with MLE in the third and fourth instars of *C. pipiens*. A: The control midgut epithelial cells' transverse section (TS); BM: basement membrane adherent to epithelial cells; N: spherical nucleus; Mv: brush border microvilli; Pm: peritrophic membrane; GL: gut lumen. Magnification strength for microscopic objects (x = 400). B-E: Transplantation staining (TS) of the midgut epithelium in larvae treated with 100% MLE showed the loss of microvilli (Mv), the peritrophic membrane (Pm), and epithelial cell death (x = 400).

*C. pipiens* larvae different histological malformations in the gastrointestinal tract, midgut, and cortex were visible in *C. pipiens*-treated larvae that had 100% MLE. These malformations included the hyperplasia of mid-gut epithelial cells, brush border crashing, ruptured membranes, and cytoplasmic masses. The untreated control larvae had both single layers of midgut epithelial cells and digestive cells. Figure 7 A–F depicts the normal brush border, cell membrane, and cytoplasm of control larvae.

### Chemical determinations

**Figure 8:**
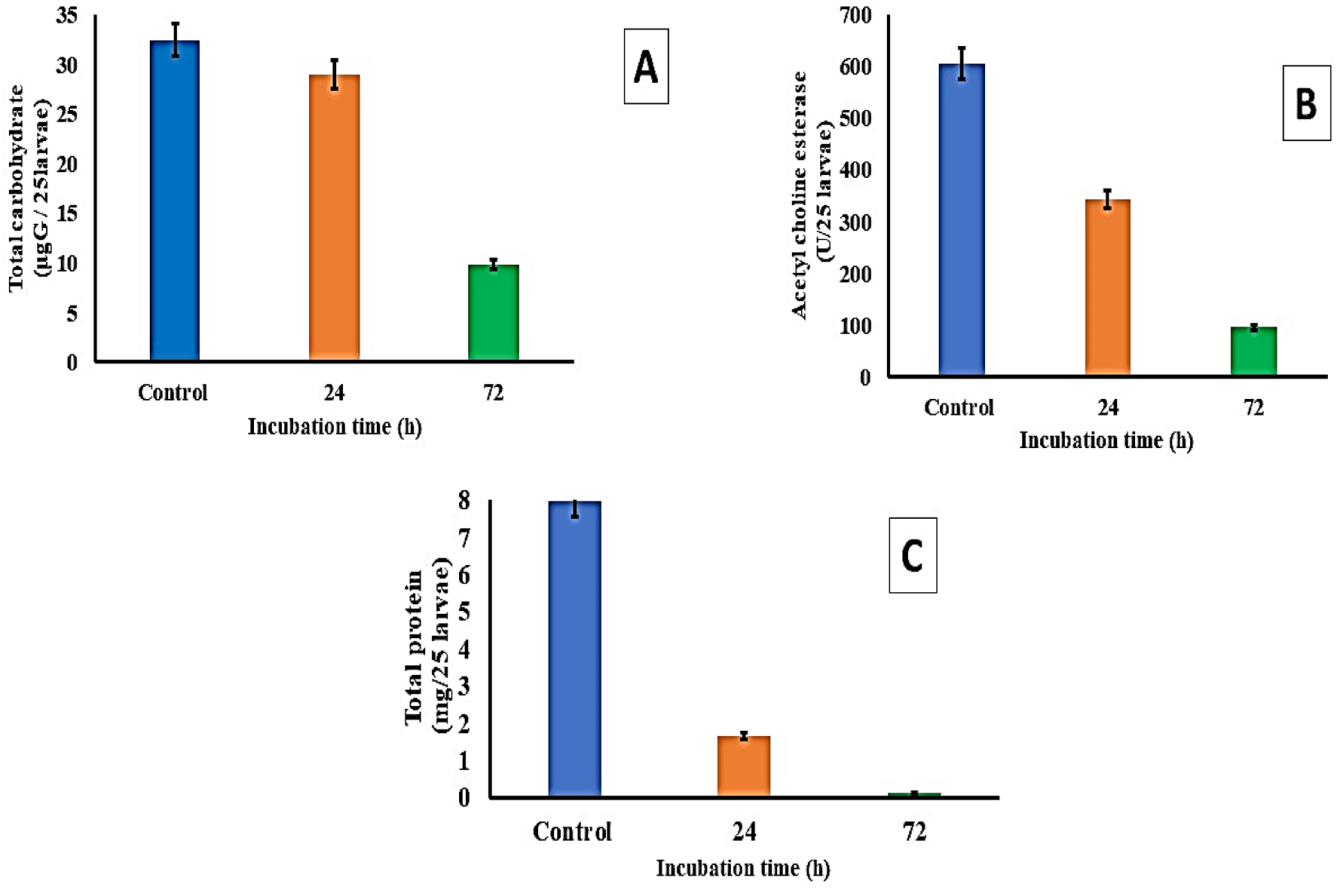
Chemical alterations for *C. pipiens*' third and fourth instars after they were exposed to 100% MLE for 72 h at 25 °C.

The third and fourth larval instars of *C. pipiens* were exposed to 100% MLE for 72h at 25 °C, and the amount of total protein, total soluble carbohydrates, and acetylcholine esterase activity were measured to determine the chemical changes that occurred. As shown in Fig. 8, it was recorded that on the third day, the protein content dropped dramatically, from the calculated control treatment value of 7.963 mg/25 larvae to just 1.661 mg/25 larvae. Between 605.10 ± 11.18 µgG / 25 larvae and 341.91 ± 12.17 µgG / 25 larvae, there was a drop in the amount of total carbohydrates. Acetylcholine esterase activity dropped from 32.44 ± 4.81 U/25 larvae to 28.96 U/25 larvae.

### Gas chromatography

GC–MS analysis is used to determine the production of active components in the mint leaf extract. The MLE comprises 39 chemical components as shown in Table 2. With a ratio of 35.92%, menthol was the largest active ingredient, followed by menthone (19.85%), D-Carvone (15.46%), Pulegone (5.0579%) and oxalic acid, isobutyl tetradecyl ester (4.05%). These phytochemicals have a variety of biological activities, including antibacterial, antifungal, and mutagenic potential, as well as anti-cancer properties. Moreover, they are phytotoxic and antioxidants.

**Table 2:** Spectral analysis of the most active compounds found in a mint (*Mentha routundifolia*) MLE sample from a GC analysis. These compounds include menthol, menthone, pulegone, and m-mentha-1,8-diene.

No.	*RT	Area (%)	Library/ID
1	5.7407	0.4819	2,3-Hexadiene, 2-methyl-
2	7.3589	2.5124	Eucalyptol
3	8.3547	0.8425	Linalool
4	9,2485	19.8519	Menthone
5	9.6974	35.9286	Menthol
6	9.7923	2.3828	m-Mentha-1,8-diene
7	9.8265	5.0579	Pulegone
8	9.9281	15.4580	D-Carvone
9	9.8765	0.7361	beta.-Bourbonene
10	10.394	2.1506	Caryphyllene
11	11.320	0.5127	Germacrene D
12	11.882	0.6562	Alpha guanin
13	11.153	0.3926	Eudesma-3,7(11)-diene
14	11.257	0.8546	Trans-Calamenene
15	11.872	0.7044	methylethenyl
16	12.0203	0.4198	Epicubenol
17	12.2268	0.5291	Cadinol
18	14.0490	4.0553	Oxalic acid, isobutyl tetradecyl ester
19	14.2264	1.5830	Pyrrolidine-3-carboxamide, 1-isopropyl-5-oxo-N-(2-thiazolyl)-
20	14.2893	3.1641	Oxalic acid, isobutyl tetradecyl ester
21	14.4095	2.2031	Oxalic acid, monoamide, n-propyl, pentadecyl ester
22	14.4553	0.6715	Oxalic acid, isobutyl heptadecyl ester
23	14.5526	3.1449	Hexadecane, 3-methyl-
24	14.6613	1.2337	Octadecane, 1-(ethenyloxy)-
25	14.6956	1.1027	Heptadecane, 9-(2-cyclohexylethyl)-
26	14.9187	1.0833	Dodecane, 1-fluoro-

## Molecular docking

### Docking simulation

**Figure 9:**
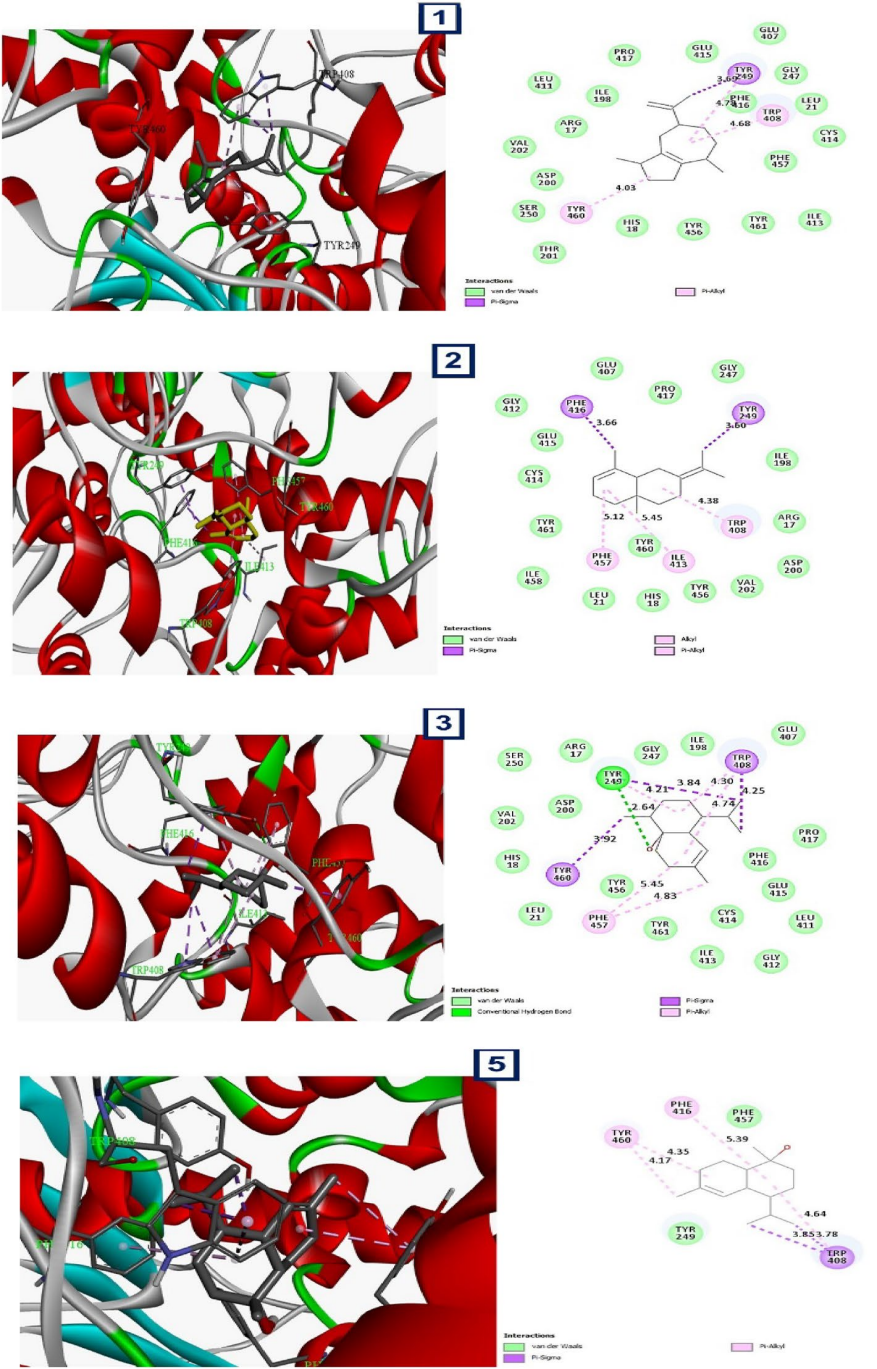

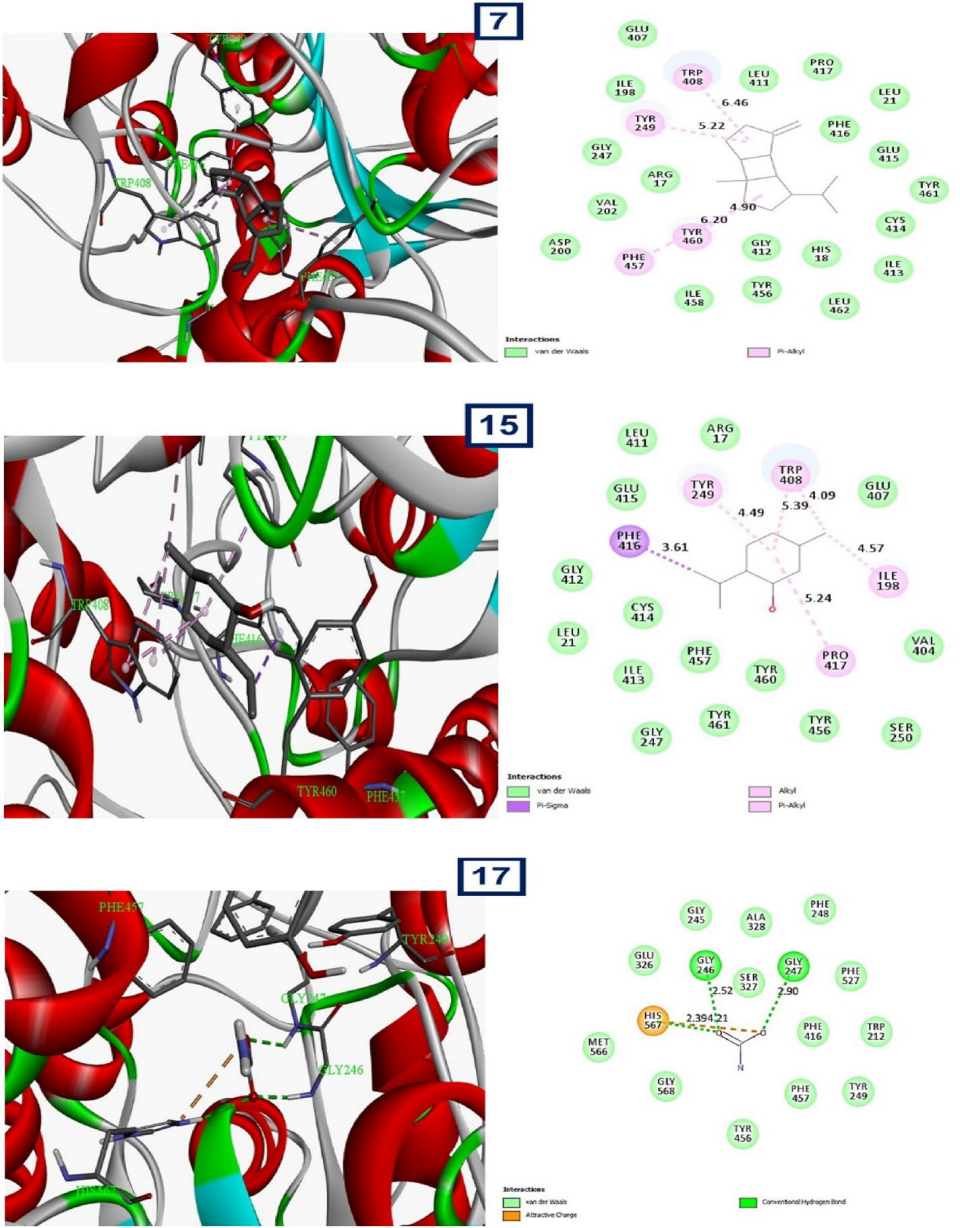
3D and 2D representations of the active sites, as seen using the BIOVIA discovery studio.

In contrast to carbamate in the first active site identified, the docking experiments showed that alpha guanine and cadinol had the highest binding affinity to both predicted active sites of *C. pipiens* acetylcholinesterase, as shown in Table 3 and Fig. 9. In active sites 2 and 1, respectively, alpha guanine displayed binding affinities of −9.3 kcal/mol and −6.8 kcal/mol followed by cadinol that showed binding affinities of −9.2 and −6.5 on active sites 1 and 2, respectively. According to these results, *C. pipiens* may be susceptible to alpha-guanine and cadinol’s potential as acetylcholinesterase inhibitors. Alpha guanine formed alkyl and pi-alkyl interactions with the receptor in active site 1, according to the interaction analysis, whereas in active site 2, it created pi-sigma interactions and van der Waals interactions with pi-alkyl. However, cadinol formed pi-alkyl interactions on both sites. The different activities observed among the other components may be due to the presence or absence of aromaticity effects and hydrogen bonding formation.

**Table 3:** Binding affinity, the total number and sites of hydrogen bonds, and pi-pi stacking formed between the ligands and the protein residues at the donepezil binding domain.

No.	Ligand compound	Binding affinity (site 1) Kcal/mol	Binding affinity (site 2) Kcal/mol	Bond interactions			Smiles code
				Bond type	Length	Residues	
1	Alpha Guaiene	−9.3	−6.8	Pi-alkyl	4.03 A	TYR 460	CC1CCC(CC2=C1CCC2C)C(=C) C
					4.68 A	TRP 408	
					4.78 A	TYR 249	
				Pi-sigma	3.69A	TYR 249	
3	Eudesma-3,7(11)-diene	−8.9	−6.8	Pi-alkyl	5.12 A	PHE 457	CC1=CCCC2(C1CC(=C(C)C)CC2)C
					4.38 A	TRP 408	
				Alkyl	5.45 A	ILE 413	
				Pi-sigma	3.60 A	TYR 249	
					3.66 A	PHE 416	
5	Epicubenol	−8.6	−6.6	Pi-alkyl	4.30 A	TRP 408	CC1CCC(C2C1(CCC(=C2)C)O)C(C)C
					4.74 A	TRP 408	
					4.21 A	TYR 249	
					5.45 A	PHE 457	
					4.83 A	PHE 457	
				H-bond	2.64 A	TYR 249	
				Pi-Sigma	3.84 A	TYR 249	
					3.78 A	TRP 408	
					3.88 A	TRP 408	
					3.92 A	TYR 460	
7	Cadinol	−9.2	−6.5	Pi-alkyl	4.35 A	TYR 460	CC1=CCCC(=C)C=CC(CC1)C(C)C
					4.17 A	TYR 460	
					5.39 A	PHE 416	
					4.64 A	TRP 408	
				Pi-alkyl	3.85 A	TRP 408	
					3.78 A	TRP 408	
13	BetaBourbonene	−9	−6.4	Pi-alkyl	6.46 A	TRP 408	CC(C)C1CCC2(C1C3C2CCC3=C)C
					4.12 A	TYR 249	
					5.05 A	TYR 460	
					5.17 A	PHE 457	
15	Menthol	−7.3	−5	Pi-alkyl	4.49 A	TYR 249	CC1CCC(C(C1)O)C(C)C
					4.30 A	TRP 408	
					3.76 A	TRP 408	
					4.32 A	TRP 408	
					4.47 A	TRP 408	
				Alkyl	4.57 A	ILE 198	
					5.24 A	PRO 417	
				Pi-sigma	3.61 A	PHE 416	
17	Carbamate (standard)	−2.7	−2.7	H-Bond	2.90 A	GLY 247	C(=O)(N)[O-]
					2.52 A	GLY 246	
				Attractive charge	2.39 A	HIS 567	
					4.21 A	HIS 567

**Figure 10:**
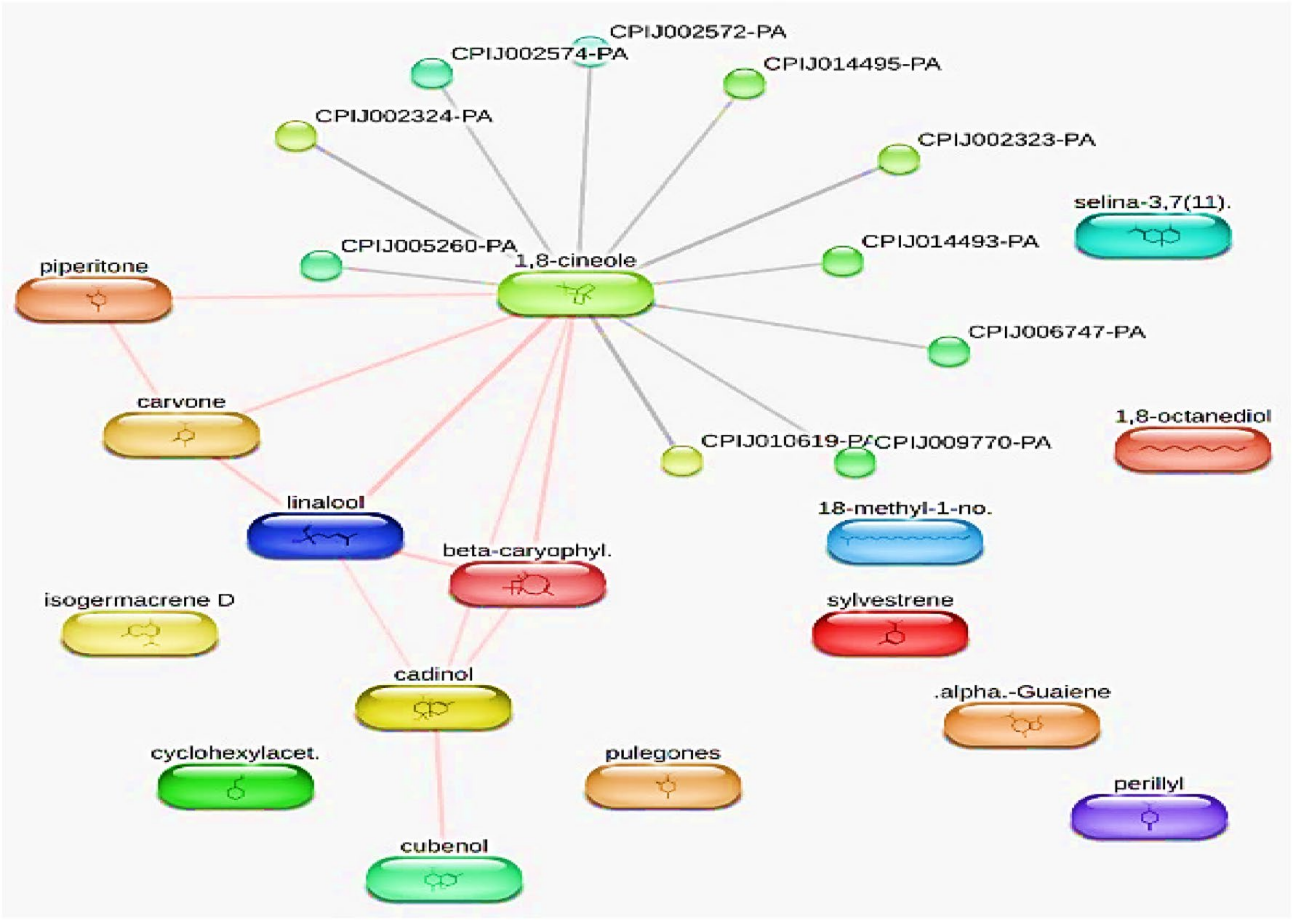
The “stitch” analysis, with generated 12 nodes representing genes or proteins and 9 edges representing interactions between them.

According to the "stitch" analysis, a network was generated with 12 nodes representing genes or proteins and 9 edges representing interactions between them, as shown in Fig. 10. The average node degree of 1.5 suggests that, on average, each node is connected to 1.5 other nodes in the network. The clustering coefficient of 0.925 indicates that the nodes tend to be highly interconnected with each other, forming tight clusters. The expected number of edges in the network is 12, and the PPI enrichment p-value of 0.848 indicates that the observed number of interactions in the network is not significantly different from what would be expected by chance. In other words, the network does not show evidence of being enriched for protein–protein interactions (PPIs). However, the functional enrichment analysis identified several overrepresented PFAM and INTERPRO protein domains in the network, which may suggest specific biological functions or pathways that are active in the system.

## Discussion

The common house mosquito, *C. pipiens* L. (Culicidae), is one of the historical lymphatic filariasis vectors in Egypt and the world. The ability of *Culex sp.* to act as a vector for viral microbes has been extensively studied, but little is known about the bacterial and fungal microbes that live in their gut. The mosquito species *C. pipiens* is widespread throughout the world and is prevalent in Egypt's urban and rural areas. It is a vector that poses a risk to human public health because it spreads many pathogens, including *B. cereus, B. anthrax, and S. warneri*^5^, which are known to cause several illnesses, including West Nile fever, Japanese encephalitis, Dengue fever, Rift Valley fever, Bancroftian filariasis, and Avian malaria^3,13^. Despite being widely distributed, very little is known about the gut bacterial symbionts of *C. pipiens*^5^. Furthermore, *S. aureus* has been and continues to be recognized as one of the most important opportunistic pathogens in humans^7,28^.

The majority of nosocomial infections, which have been linked to continuous increases in healthcare costs, have also been caused by *S. aureus* antimicrobial resistance. Antibiotics are frequently administered to hospitalized patients with nosocomial infections, which promotes the colonization and infection of multidrug-resistant microorganisms^29^. It is unfortunately common for MDR pathogen infections to be associated with high rates of morbidity and mortality, making it essential to quickly identify any mutant isolates and assess their susceptibility profiles to properly direct treatment.

In this study, we evaluated the MDR ubiquity and antibiotic susceptibility patterns of the most recovered pathogens of *S. aureus* obtained from *C. pipiens* L., insect midgut specimens, around three hospitals located in Cairo governorate, Egypt. A susceptibility test for antibiotics was performed on 100 isolates. Isolates with multidrug resistance to two or more commonly used commercial antibiotics^30^. All MDR *S. aureus* isolates (except five) were shown to be responsive to Mint leaf at MIC ≤ 700 μg/mL in our investigation. Additionally, the data from this study showed that, despite several other documented cases from patients in this area, the MLE has good antibacterial activity when used in a variety of concentrations against isolated MDR *S. aureus*.

Multiple factors, including variations in susceptibility testing procedures and extraction processes, may be responsible for these results. It states that the polysaccharides in the mint extract have medicinal properties, including immunostimulant, anti-inflammatory, wound healing, stimulation of hematopoiesis, and anti-oxidant effects. Mint contains many pharmacologically active substances, such as menthone and polygons. *Anopheles culicifacies*, which are responsible for 70–75% of malaria transmission in the northern rural areas of India, were found to be strongly attracted to the essential mint leaf. According to our research, the high mortality action of MLE could be attributed to its active ingredients, including menthol, Neither the full power of natural products nor their exact mechanisms of action are fully understood by us.

Scientists from all over the world are becoming more and more interested in natural antimicrobial phytochemicals, and they are working to understand these ingredients' mechanisms in depth. To provide more proof, it is crucial to continue these studies with a sizable sample. Such work is crucial for the region's overall health as well as the local community. Mint has potent larvicidal and antibacterial properties that are effective against *C. pipiens* and the *S. aureus* that reside in their midgut.

According to the World Health Organization (WHO), 80% of people in developing countries^3^ used traditional therapies. As they play a significant role as antimicrobial and anti-inflammatory agents, they rely on the use of medicinal herbal plant extracts to treat various infectious diseases^4^.

Since it was discovered that mint leaf extract has inhibitory potentials against a variety of pathogenic bacteria, including *Escherichia coli*, *Bacillus subtilis*, *Salmonella typhi*, *Pseudomonas* sp., and *Klebsiella epidermidis*^31^, it is the most significant antibacterial compound used by many nations in burn treatments. It was demonstrated that mint had high antibacterial activity in a study of its inhibitory activities and MIC. Furthermore, a few of the active components in mint are commercially obtainable and are used as insecticidal^16^ and antimicrobial agents to manage pest insects and plant diseases16. Based on the chemical analysis of mint extracts, the majority of mint species contained 1,8-cineole, menthol, menthone, carvone, and pulegone. Numerous insects are resistant to the insecticidal effects of mint species, according to studies^32^.

Commercial antibiotics such as ciprofloxacin, gentamycin, and tetracycline are frequently administered in Egypt as standard treatments for *S. aureus*-caused burn wound infections^33^. In *S. aureus* burn wound patients, the use of these antibiotics increases the risk of MDR^34^. Moreover, this resistance presents serious therapeutic challenges for *S. aureus* treatments.

According to the study's findings, alpha guanine had a strong binding affinity for the two identified active sites of *C. pipiens*' acetylcholinesterase, with binding affinities of −6.8 kcal/mol for active site 1 and −9.3 kcal/mol for active site 2. These findings imply that acetylcholinesterase in *C. pipiens* may be susceptible to inhibition by alpha-guanine.

Upon further examination of the interactions between alpha-guanine and the active sites, it was discovered that active site 1's ligand formed alkyl and pi-alkyl interactions with the receptor, with an average distance of 5 angstroms. Alkyl interactions occur between non-reactive, homologous carbon groups in organic molecules and are typically weak interactions. Pi-alkyl interactions occur between aromatic and aliphatic groups and are characterized by the overlap of the pi-electron density of the aromatic ring with the electron density of the alkyl group. These interactions imply that the weak, non-covalent van der Waals interactions between alpha guanine and the receptor may explain some of the binding affinity of the compound.

In active site 2, the ligand formed pi-sigma and van der Waals interactions with pi-alkyl, with an average distance of 4 angstroms. Pi-sigma interactions take place when the electron density of a sigma bond interacts with the pi-electron density of an aromatic ring, and they are distinguished by the perpendicular orientation of the two interacting components. Van der Waals interactions are weak interactions that occur between atoms or molecules due to fluctuations in their electron density. Pi-alkyl interactions refer to the interaction between an aromatic ring and an alkyl group. These interactions suggest that alpha guanine may form a stable complex with Acetylcholinesterase in *Culex pipiens* through a combination of pi-sigma and van der Waals interactions.

## Data Availability

The data that support the findings of this study are available from the corresponding author upon reasonable request. Raw sequencing files and associated metadata have been deposited at NCBI's Sequence Read Archive (accession OQ766965). https://www.ncbi.nlm.nih.gov/nucleotide/OQ766965.1*. S. aureus* ATCC 29,737 was from the ATCC collection https://www.atcc.org/products/29737. Mega 11 software was used from https://www.megasoftware.net/.
